# Nutrient Partitioning and Stoichiometry in Unburnt Sugarcane Ratoon at Varying Yield Levels

**DOI:** 10.3389/fpls.2016.00466

**Published:** 2016-04-20

**Authors:** José M. Leite, Ignacio A. Ciampitti, Eduardo Mariano, Michele X. Vieira-Megda, Paulo C. O. Trivelin

**Affiliations:** ^1^Department of Soil Science, University of São PauloPiracicaba, Brazil; ^2^Department of Agronomy, Kansas State University, ManhattanKS, USA; ^3^Laboratory of Stable Isotopes, University of São PauloPiracicaba, Brazil; ^4^Department of Agriculture, State University of Montes ClarosJanaúba, Brazil

**Keywords:** *Saccharum* spp., nutrient uptake, nitrogen, phosphorus, potassium, biomass, internal efficiencies

## Abstract

Unraveling nutrient imbalances in contemporary agriculture is a research priority to improve whenever possible yield and nutrient use efficiency in sugarcane (*Saccharum* spp.) systems while minimizing the costs of cultivation (e.g., use of fertilizers) and environmental concerns. The main goal of this study was therefore to investigate biomass and nutrient [nitrogen (N), phosphorus (P), and potassium (K)] content, partitioning, stoichiometry and internal efficiencies in sugarcane ratoon at varying yield levels. Three sites were established on highly weathered tropical soils located in the Southeast region of Brazil. At all sites, seasonal biomass and nutrient uptake patterns were synthesized from four sampling times taken throughout the sugarcane ratoon season. In-season nutrient partitioning (in diverse plant components), internal efficiencies (yield to nutrient content ratio) and nutrient ratios (N:P and N:K) were determined at harvesting. Sugarcane exhibited three distinct phases of plant growth, as follows: lag, exponential–linear, and stationary. Across sites, nutrient requirement per unit of yield was 1.4 kg N, 0.24 kg P, and 2.7 kg K per Mg of stalk produced, but nutrient removal varied with soil nutrient status (based on soil plus fertilizer nutrient supply) and crop demand (potential yield). Dry leaves had lower nutrient content (N, P, and K) and broader N:P and N:K ratios when compared with tops and stalks plant fractions. Greater sugarcane yield and narrowed N:P ratio (6:1) were verified for tops of sugarcane when increasing both N and P content. High-yielding sugarcane systems were related to higher nutrient content and more balanced N:P (6:1) and N:K (0.5:1) ratios.

## Introduction

Brazil is the world’s largest sugarcane (*Saccharum* spp.) producer, with approximately 9 million hectares (ha) cultivated for biofuel, sugar, and electricity production (UNICA, 2015). In the last decade, cultivated sugarcane area in Brazil increased by about 4 million ha, primarily in the Southeast and Midwest regions. The latter expansion was explained by the significant increase in bioethanol consumption by flex-fuel light-duty vehicles (Adami et al., 2012). The increase in cropland area is growing in parallel with technology investments implemented by sugarcane growers, such as machinery for planting and harvest operations. Due to environmental, agronomic, social, and economical reasons, manual harvest with prior burning of sugarcane has been replaced by harvesters, where dry leaves and tops (developing leaves and meristem) are now left over the soil surface, representing an input from 10 to 20 Mg ha^-1^ year^-1^ of dry biomass (Fortes et al., 2013; Leal et al., 2013; Trivelin et al., 2013). This new harvest system has been called “green cane management system,” where ∼80% of the Brazilian sugarcane fields previously harvested by hand with burning are currently harvested without burning by harvesters (Leal et al., 2013). In São Paulo, largest sugarcane-producing state, the crop is cultivated in 4.7 million ha, and more than 85% of the total is mechanically green harvested (UNICA, 2015).

The adoption of the green cane management system coupled with other management practices (e.g., precision agriculture tools) may modify nutrient requirements for modern sugarcane cultivars. Traditional approaches to the mineral nutrition of sugarcane included a strong emphasis on yield with a focus on a balanced nutrition, economic productivity, and quality (Kingston, 2014). Balanced nutrition implies that all essential nutrients needed for proper growth and ripening of sugarcane are available (Kingston, 2014). Nitrogen (N) and potassium (K) are demanded in large amounts by the crop, ranging from 150 to 400 kg ha^-1^ (Silva and Casagrande, 1983; Shukla et al., 2009; Oliveira, 2011; Mariano et al., 2016) for sugarcane yield varying from 70 to 180 Mg ha^-1^. Nitrogen is a major nutrient for plant growth and tillering processes (Kingston, 2014), while K plays a key role on osmoregulation, which is important for cell extension, stomata movement, and enzyme activation (Epstein and Bloom, 2005; Shukla et al., 2009; Kingston, 2014). Phosphorus (P) is also essential for tillering, root and shoot growth (Kingston, 2014). In highly weathered tropical soils, usually characterized by low nutrient availability, monitoring the fertility status and fertilizer (NPK) application are essential practices to achieve and sustain high sugarcane yield levels (Franco et al., 2011; Oliveira, 2011; Trivelin et al., 2013).

For sugarcane, the nutrient ratio concept (also termed as nutrient stoichiometry) is not a new tool for diagnosing nutrient imbalances. Beaufils and Sumner (1976) developed the DRIS to evaluate N, P, and K requirements of sugarcane irrespective of plant age. Imbalances of these nutrients can be detected by means of DRIS before a deficiency can be diagnosed securely by means of threshold values (Beaufils and Sumner, 1976; Meyer, 1981). However, nutrient ratios investigated were unstable. Nutrient ratios (N:P and N:K) changed during the stage of sugarcane development (Meyer, 1981). This occurs, because there is a complex interaction and dynamics of these elements within the soil-plant system (Meyer, 1981; Shukla et al., 2009; Mariano et al., 2016). Besides this complexity, these studies did not properly address the role of the nutrient ratios relative to the sugarcane yield. Second, previous research on nutrient ratios (N:P and N:K) for sugarcane probably were carried out in fields harvested with prior burning, a different condition to the current and future scenario. More than 70% of the total organic matter and nutrients contained in the sugarcane straw are emitted to the atmosphere via burning prior manual harvest (Mitchell et al., 2000). Therefore, the straw layer left over the soil surface when the crop is mechanically harvested without burning represents a significant input of organic C and nutrients to the soil-plant system, which can result in long-term benefits for the sustainability (via C sequestration) and nutritional requirements (by decreasing fertilizer needs) of sugarcane systems (Trivelin et al., 2013). Likely, nutrient ratios correlate with sugarcane yield in the green cane management system, but further investigations are required for examining this new research area.

Brazil is one of the largest consumers of fertilizer, with a nutrient demand expected to rise in the foreseeable future (IPNI, 2015). However, the country strongly depends on the international fertilizer market, especially for N (78%) and K (90%). A feasible strategy to reduce this dependency is to increase NUE, measured as sugarcane yield to nutrient content ratio (so called “physiological efficiency”). Management options for improving NUE from a cropping system perspective must also consider both NUE components: nutrient recovery and physiological efficiencies (Cassman et al., 2002; Ciampitti and Vyn, 2012). In sugarcane, little is known about NUE for green cane management systems. Vieira-Megda et al. (2015) investigated the effect of fertilizer N sources and rates on sugarcane productivity, without documenting changes in biomass accumulation. Similar results were reported by Otto et al. (2013), where among six sugarcane N-response trials, only one site showed high yield response (>25%) to fertilizer N. Probably, lack of adequate balance on nutrient ratios (N:P and N:K) can act as yield-limiting factor in the green cane management system. Nutrient balances can help in closing yields gaps and improve NUE. Therefore, plant nutrient ratios are valuable tool for diagnosing crop nutrient imbalances (Ciampitti and Vyn, 2014). The aim of this study was to investigate and synthesize information on biomass and nutrient (N, P, and K) content, partitioning, stoichiometry, and internal efficiency indexes in sugarcane ratoon at varying yield levels at three site-years.

## Materials and Methods

### Site Characteristics and Sugarcane Genotypes

Three N-response trials for sugarcane fertilization were carried out in the state of São Paulo, Southeast region of Brazil. Two site-years were performed during the 2009/2010 growing season (Sites 1 and 2), while the remaining experiment (Site 3) was conducted in the 2012/2013 season. At all sites, experiments were established in the first ratoon cycle, and the plant cane (previous cycle) was harvested without burning the residues, leaving a straw layer (formed by dry leaves and tops) over the soil surface. Conventional tillage (plowing, disking, harrowing, and furrowing) and soil correction (application of lime and gypsum) were performed, on average, every 6 years at all sites. Site 1 was located in Piracicaba (22°35′S; 47°37′W), in a Typic Hapludox soil (Soil Survey Staff, 2014). Plant cane (cultivar SP89-1115, released by Copersucar) was mechanically green harvested in May 2009, at 433 DAP. Site 2 was located in Santa Cruz das Palmeiras (21°47′S; 47°11′W), in a Typic Eutrustox soil (Soil Survey Staff, 2014). Plant cane (cultivar SP81-3250, released by Copersucar) was also mechanically green harvested in June 2009 (456 DAP). Further details related to soil characteristics and fertilizer and by-product management history for Sites 1 and 2 can be found in Mariano et al. (2015) and Vieira-Megda et al. (2015). Site 3 was located in Severínia (20°46′S; 48°45′W), in a Typic Eutrustox (Soil Survey Staff, 2014). The cultivar planted was RB855453 (released by Ridesa), and the plant cane was harvested without prior burning in August 2012, at 442 DAP. As related to the genotypes, cultivar SP89-1115 is marketed as early ripening, average for yield potential, sucrose and fiber levels; cultivar SP81-3250 has a middle ripening, with high sugarcane yield potential, sucrose and fiber levels; and cultivar RB855453 is early ripening, but also with high yield potential and sucrose, and average fiber levels (Tasso Júnior, 2007; Ridesa, 2010).

### Soil Characterization and Experimental Setup

Before the onset of the field trial, four composite soil samples (obtained by combining and mixing nine individual samples into a single sample per block) were taken at 20-cm soil depth intervals until 100-cm soil layer for soil physicochemical characterization. Soil pH was determined with 0.01 mol L^-1^ CaCl_2_ (ratio of 1:2.5 for soil and solution – w/v; van Raij et al., 2001). Soil organic C was determined by wet oxidation following the Walkley–Black procedure (Nelson and Sommers, 1996). Available P, K, calcium (Ca), and magnesium (Mg) were extracted by ion-exchange resins, and then quantified by colorimetric (P), flame photometric (K), and atomic absorption spectroscopic (Ca and Mg) methods (van Raij et al., 2001). Sulfur (SO_4_^-2^-S) was extracted with Ca(H_2_PO_4_)_2_ solution and determined by turbidimetry, while exchangeable aluminum (Al) was extracted with KCl solution and then determined by titration (van Raij et al., 2001). CEC at pH 7.0 was measured by summation of exchangeable cations (K, Ca, and Mg) and potential acidity (H + Al). Base saturation was calculated by dividing the summation of exchangeable cations by CEC, and then multiplied by 100. Clay content was determined by the densimeter method (Gee and Bauder, 1986). Physicochemical soil properties are displayed in **Table 1**.

**Table 1:** Selected physicochemical soil properties at the 0- to 100-cm soil layer before the onset of field trials cropped with sugarcane at three sites, all located in the Southeast region of Brazil.

Depth cm	pH	SOC^a^ g dm^-3^	P	S	K	Ca	Mg	Al	CEC^b^	BS^c^ %	Clay g kg^-1^
						
			mg dm^-3^		mmol_c_ dm^-3^						
**Site 1 (Typic Hapludox soil^d^)**								
0–20	4.6	23	29	33	4.3	13	6	5	95	24	510
20–40	4.6	19	29	50	4.6	29	12	5	126	36	530
40–60	4.3	19	5	95	4.9	13	8	11	124	21	500
60–80	4.1	14	2	107	5.5	8	6	13	117	17	580
80–100	4.2	13	1	105	4.0	9	8	13	109	19	530
**Site 2 (Typic Eutrustox soil^d^)**								
0–20	5.5	17	8	27	0.7	49	13	0	91	69	630
20–40	4.8	13	16	92	0.3	33	10	2	85	51	630
40–60	4.6	10	2	116	0.2	19	8	4	69	39	640
60–80	4.7	8	1	117	0.2	16	7	2	57	41	660
80–100	4.9	7	1	113	0.1	16	7	1	57	40	660
**Site 3 (Typic Eutrustox soil^d^)**								
0–20	5.4	9	28	12	3.0	18	9	1	49	61	299
20–40	5.3	7	33	30	1.4	15	9	3	47	54	226
40–60	5.2	5	3	47	1.0	13	10	2	41	58	252
60–80	5.2	5	2	50	0.8	14	10	1	39	64	298
80–100	5.2	4	2	49	0.8	12	11	1	33	71	275


*^a^Soil organic C.*

*^b^Cation exchange capacity at pH 7.0.*

*^c^Base saturation.*

*^d^Classification refers to the Soil Taxonomy (Soil Survey Staff, 2014).*

**FIGURE 1:**
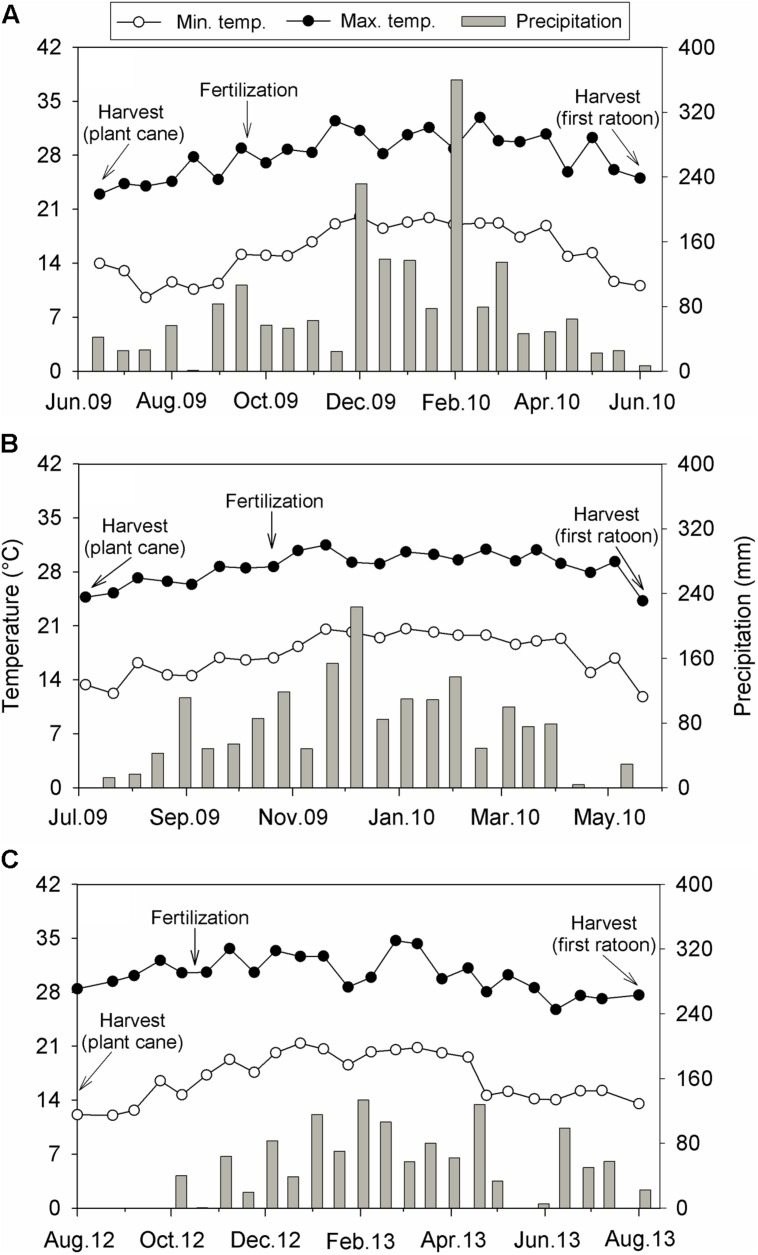
**Biweekly precipitation and mean biweekly minimum and maximum air temperatures during a sugarcane growing season (first ratoon cycle) conducted at Site 1 **(A)**, Site 2 **(B)**, and Site 3 **(C)**, all located in the Southeast region of Brazil**.

The experimental design within each location was a randomized complete block with four (Sites 1 and 2) or five (Site 3) replications. At Site 1, treatments were fertilizer N sources [ammonium chloride, ammonium nitrate, calcium ammonium nitrate, organo-mineral fertilizer, urea, and control (no-N added)], applied at a rate of 100 kg N ha^-1^. For Site 2, in addition to the above-cited treatments applied at Site 1, ammonium sulfate (100 kg N ha^-1^) was also used. At Site 1, plots were fertilized with 75 kg K ha^-1^, while 125 kg K ha^-1^ and 15 kg P ha^-1^ were added at Site 2. The sources of K and P used at both sites were KCl and triple superphosphate, respectively. At both sites, fertilizers were manually applied over the straw layer, in a band at 20 cm away from the plant row. For Site 3, treatments used were rates of HSS (35 g organic C kg^-1^; 115, 230, and 460 kg HSS ha^-1^), N (25, 50, and 100 kg N ha^-1^) and specific interactions between both factors (115 kg HSS ha^-1^+ 25 kg N ha^-1^, 230 kg HSS ha^-1^+ 50 kg N ha^-1^, and 460 kg HSS ha^-1^+ 100 kg N ha^-1^). A control treatment (without addition of HSS and N) was also used. Humic substances were composed by fulvic and humic acids, both extracted and purified from peat, whereas N was applied as urea. The HSS and HSS + N treatments were applied directly to sugarcane leaves (foliar application), while N rates were banded added over the straw layer, at 20 cm away from the plant row. Treatments were added on September 2009, October 2009, and October 2012 at Sites 1, 2, and 3, respectively. Phosphorus and K recommendations were defined based on soil test methods (**Table 1**), whereas target yield concept was employed to predict fertilizer N requirements (Spironello et al., 1997). Biweekly precipitation and mean biweekly minimum and maximum air temperatures recorded at each site are displayed in **Figure 1**.

### Biomass and Plant Nutrient Content Measurements

Plant measurements followed similar protocols at all sites, and a complete description can be found in Mariano et al. (2016). Briefly, plants were randomly collected from 3-m plant row (∼30 plants) in each experimental plot, and then separated into stalks, dry leaves, and tops at all four sampling times (comparable growth stages in all sites). In our study, tops refer to developing (green) leaves from the stalk breakpoint to the apical meristem. Sampling dates of plant biomass were performed at 139, 178, 235, and 330 DAH for Site 1; at 133, 170, 227, and 331 DAH for Site 2; and at 145, 198, 290, and 380 DAH for Site 3. Fresh samples were weighed and then ground in a forage grinder. Subsamples of each plant component were oven dried at 65°C until achieving constant weight, then weighed for dry weight determination, and ground to pass through a 0.5-mm sieve in a Wiley mill. Total N, P, and K concentration (g kg^-1^) in plant tissues were determined by steam distillation (Kjeldahl), gravimetry, and flame emission photometry, respectively (AOAC International, 2006). Total aboveground biomass accumulation (expressed in Mg ha^-1^) was determined by the summation of all plant components collected in each sampling date. Total N, P, and K content (kg ha^-1^) in each plant component was calculated by multiplying each nutrient concentration by its respective biomass value (in dry weight). Final aboveground biomass and nutrient content were estimated based on the last sampling date performed at each site. Sugarcane yield (Mg ha^-1^) was estimated based on the fresh matter of stalks collected from 3-m plant row in the last sampling date.

### Biomass and Nutrient Partitioning

Seasonal biomass partitioning in dry leaves, stalks, and tops of sugarcane collected along the ratoon cycle was fitted using a sigmoid equation for estimating the crop growth modeling (Mariano et al., 2016). The following equation (Eq. 1) was then used to predict seasonal biomass within each plant component:

$$Y=\frac{Y_{\max}}{\left\{1+\exp-\left[(DAH-A)/B\right]\right\}}$$

where *Y* is the aboveground biomass (Mg ha^-1^); *Y*_max_ is the maximum aboveground biomass (Mg ha^-1^) from harvest of the plant cane to harvest of the first ratoon; DAH is days after harvest of plant cane; *A* and *B* are constants.

Nutrient (N, P, and K) partitioning among each plant component along the ratoon cycle was fitted using a Gaussian equation (Eq. 2) as follows:

$$Y=Y_{0}+A\times \exp\left\{-0.5\times {\left[\frac{DAH-B}{C}\right]}^{2}\right\}$$

where *Y* is the aboveground nutrient (N, P, and K) content (kg ha^-1^); *Y*_0_ is the intercept; DAH is days after harvest of plant cane; *A*, *B*, and *C* are constants.

Thermal time was expressed as GDDs, which are calculated as accumulation of daily mean temperature values [(min. temperature + max. temperature)/2] minus a base temperature (*t*_b_), below which growth is assumed not to occur. In this study, *t*_b_ was taken as 12°C, an appropriate value for sugarcane, according to Allison et al. (2007). For terminology purposes, the term “aboveground” will be used throughout the text to refer to biomass and nutrient content in the following plant components: tops, dry leaves, and stalks.

### Harvest Index and Nutrient and Reciprocal Internal Efficiencies

Harvest index was calculated as the stalks to the aboveground biomass ratio (all determined in dry weight basis) obtained in the last sampling date performed at each site. Similarly, nutrient HI for N, P, and K were determined as the nutrient content in the stalk as related to the total content in the aboveground plant fraction. NIE (kg stalks kg^-1^ nutrient) was defined as the final sugarcane yield (converted from Mg ha^-1^ to kg ha^-1^) produced per kg of nutrient content (N, P, and K) in aboveground biomass component according to Ciampitti and Vyn (2012). Therefore, the following equation (Eq. 3) was then used for the NIE calculation:

$$NIE=\left(\frac{SY}{WNC}\right)$$

where NIE is nutrient internal efficiency; SY is the sugarcane yield (kg ha^-1^); and WNC is the whole-plant (aboveground) nutrient (N, P, and K) content (kg ha^-1^).

In addition, the RIE (kg nutrient Mg^-1^ stalk) was calculated according to Ciampitti and Vyn (2012), following the equation (Eq. 4):

$$RIE=\left(\frac{WNC}{SY}\right)$$

where RIE is reciprocal internal efficiency; WNC is the whole-plant (aboveground) nutrient (N, P, and K) content (kg ha^-1^); and SY is the sugarcane yield (Mg ha^-1^)

### Statistical Analysis

Although there were consistent differences among the three N-response sites in terms of experimental conditions (e.g., nutrient rates applied, fertilizer sources, cultivars planted, etc.), this study performs a synthesis analysis to provide general information on nutrient content and partitioning, based on varying sugarcane yield levels, regardless of evaluated factors within each site. This approach was previously used by Setiyono et al. (2010) for corn. For all the parameters studied, descriptive statistics was determined through calculating mean, standard deviation, minimum, maximum, and 25–75% quartile. The equations used to predict seasonal biomass and nutrient partitioning (Eqs. 1 and 2) among plant components (stalks, dry leaves, and tops) were chosen based on their higher *R*^2^ values than other functions tested. The relationship between the sugarcane yield versus the aboveground nutrient content and stalk nutrient content for N, P, and K was assessed through non-linear regressions. The model adjusted was validated utilizing previously published studies (Shukla et al., 2009; Oliveira et al., 2010; Schultz et al., 2010) as to demonstrate the fitness and robustness of model calibration. The relationship between N content versus P and K content were also performed aimed to evaluate the N:P and N:K ratios within each plant component. All fitted regressions were determined using the SigmaPlot graphing software (version 11.0, 2008, Systat Software Inc., San Jose, CA, USA). The aboveground biomass accumulation, nutrient content, NIE, and RIE values obtained from all sites were stratified into diverse sugarcane yield ranges (<80, 80–100, 100–120, 120–140, 140–160, and >160 Mg ha^-1^).

## Results

### Weather Conditions

At the three sites, precipitation totals during the sugarcane growing season (equivalent to 12 months) were above the local average (**Figure 1**). At Site 1, precipitation reached 1,862 mm and was 1.5-fold higher than the 81-year average (1,230 mm; **Figure 1A**); precipitation at Site 2 totaled 1,691 mm and was 1.3-fold greater than the 29-year average (1,330 mm; **Figure 1B**); at Site 3, cumulative precipitation (1,310 mm) was slightly superior than the 17-year average (1,266 mm; **Figure 1C**). The air temperatures recorded at each site (**Figure 1**) were similar to historical average. At Site 1, minimum air temperature varied from 9.5 to 20.0°C, while maximum air temperature ranged from 24.2 to 32.9°C. At Site 2, minimum air temperature ranged from 11.8 to 20.6°C, while maximum air temperature varied from 24.2 to 31.5°C. Lastly, at Site 3, minimum air temperature varied from 12.0 to 21.4°C, and maximum air temperature ranged from 25.7 to 34.7°C.

### Biomass, Nutrient Content, and Harvest Index

A broad range in aboveground sugarcane biomass was recorded across all sites (**Table 2**). Sugarcane yield ranged from 67 to 211 Mg ha^-1^, averaging 125 Mg ha^-1^. Aboveground biomass ranged from 16 to 106 Mg ha^-1^, averaging 57 Mg ha^-1^. Stalk biomass ranged from 9 to 66 Mg ha^-1^, averaging 37 Mg ha^-1^, while tops mass ranged from 2 to 30 Mg ha^-1^ and averaged 11 Mg ha^-1^. Dry leaves biomass ranged from 4 to 20 Mg ha^-1^, with an average of 9 Mg ha^-1^. Nutrient content within each plant component also had a broad variation (**Table 2**). The content of N, P, and K in the stalks ranged from 32 to 168 kg ha^-1^, from 5 to 57 kg ha^-1^, and from 26 to 713 kg ha^-1^, respectively. The content of N, P, and K in the dry leaves ranged from 19 to 77 kg ha^-1^, 0.6 to 4.9 kg ha^-1^, and 2 to 96 kg ha^-1^, respectively. Lastly, the content of N, P, and K in the tops ranged from 17 to 225 kg ha^-1^, 1 to 26 kg ha^-1^, and 75 to 396 kg ha^-1^, respectively. As related to the aboveground plant fractions (dry leaves + tops + stalk), N content ranged from 86 to 425 kg N ha^-1^, averaging 197 kg N ha^-1^, while P content ranged from 10 to 79 kg P ha^-1^, averaging 32 kg P ha^-1^ (**Table 2**). Aboveground K content followed a similar trend related to the N content (**Table 2**), with values ranging between 159 and 866 kg K ha^-1^, averaging 469 kg K ha^-1^. The biomass HI had a narrow range, varying from 0.55 to 0.73 across all evaluated sites (**Table 2**). In contrast, nutrient HI showed a broad range, varying from 0.31 to 0.64 for N, 0.38 to 0.81 for P, and 0.12 to 0.82 for K. Detailed results about biomass accumulation, nutrient content, and HI for each specific treatment within each site can be found in **Supplementary Table 1**.

**Table 2:** Descriptive statistics related to sugarcane yield, aboveground biomass and its components (stalks, dry leaves, and tops), content of nitrogen (N), phosphorus (P), and potassium (K) allocated in each plant component, HI and HI of N, P, and K for sugarcane (first ratoon cycle) across three sites located in the Southeast region of Brazil (*n* = 102).

Parameter	Unit	Mean	*SD*^a^	Minimum	25% Q^b^	Median	75% Q^c^	Maximum
Sugarcane yield	Mg ha^-1^	125	42	67	84	119	164	211
Aboveground biomass	Mg ha^-1^	57	28	16	29	46	84	106
Stalk biomass	Mg ha^-1^	37	18	9	20	31	54	66
Dry leaves biomass	Mg ha^-1^	9	3	4	6	8	11	20
Tops biomass	Mg ha^-1^	11	7	2	4	8	18	30
Aboveground N content	kg ha^-1^	197	73	86	135	175	254	425
Aboveground P content	kg ha^-1^	32	18	10	14	27	45	79
Aboveground K content	kg ha^-1^	469	169	159	366	502	578	866
Stalk N content	kg ha^-1^	90	31	32	64	88	112	168
Stalk P content	kg ha^-1^	19	12	5	8	16	26	57
Stalk K content	kg ha^-1^	266	153	26	202	275	346	713
Dry leaves N content	kg ha^-1^	34	11	19	27	33	39	77
Dry leaves P content	kg ha^-1^	2.0	1.0	0.6	1.2	1.7	2.5	4.9
Dry leaves K content	kg ha^-1^	26	18	2	13	19	36	96
Tops N content	kg ha^-1^	74	45	17	33	54	110	225
Tops P content	kg ha^-1^	11	7	1	5	8	17	26
Tops K content	kg ha^-1^	177	58	75	133	171	209	396
Harvest index^d^	–	0.65	0.04	0.55	0.64	0.66	0.68	0.73
N harvest index^e^	–	0.46	0.06	0.31	0.41	0.46	0.50	0.64
P harvest index^e^	–	0.57	0.08	0.38	0.51	0.57	0.62	0.81
K harvest index^e^	–	0.52	0.19	0.12	0.45	0.53	0.65	0.82


*^a^Standard deviation.*

*^b^First quartile.*

*^c^Third quartile.*

*^d^Calculated as the stalks to the aboveground biomass ratio.*

*^e^Calculated as the nutrient content in the stalk as related to the total content in the aboveground plant fraction.*

### Biomass and Nutrient Partitioning

**FIGURE 2:**
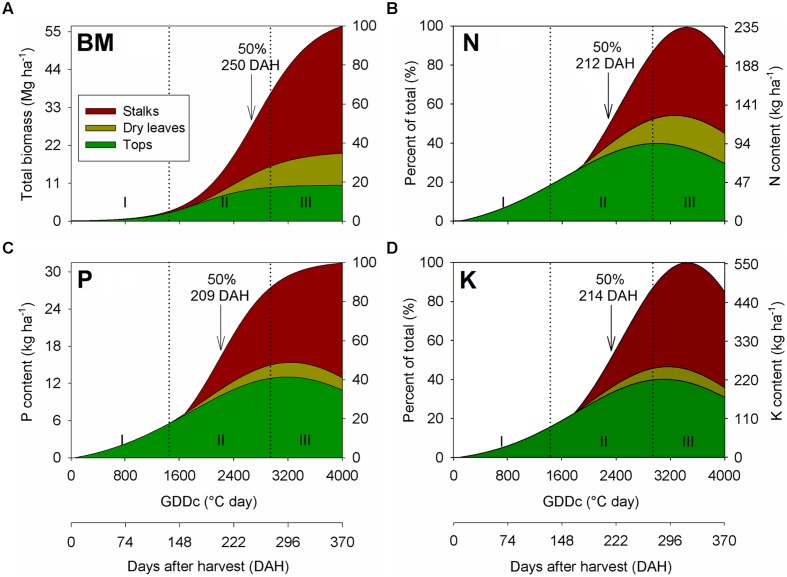
**Seasonal aboveground biomass (BM; **A**), nitrogen (N; **B**), phosphorus (P; **C**), and potassium (K; **D**) accumulation and partitioning in stalks, dry leaves, and tops of sugarcane during a first ratoon growing season, in overall for all three site-years located in the Southeast region of Brazil.** Arrows indicate when sugarcane BM and nutrient accumulation achieved 50% relative to its final content at harvest time.

Sugarcane biomass accumulation throughout the ratoon cycles was characterized by a sigmoid pattern (**Figure 2A**). The non-linear growth regression equation included data from all sites, and three distinct growth phases were identified, as follows: (1) Phase I (lag phase): from ∼0 to 135 DAH (0 to 1450°C GDDs), this period is marked by a slow plant growth, accumulating ∼10% of the final aboveground biomass relative to harvest; (2) Phase II (exponential–linear phase): from ∼135 to 270 DAH (1450 to 2800°C GDDs), this period was characterized by a rapid growth that produced ∼65% of the final aboveground biomass relative to harvest; and (3) Phase III (stationary phase): from ∼270 to 370 DAH (2800 to 4000°C GDDs), this period exhibited a declined growth, representing ∼25% of the final aboveground biomass at harvest. As related to the nutrient uptake, 50% of the total N, P, and K content occurred at 212 (2259 GDDs), 209 (2292 GDDs), and 214 DAH (2314 GDDs), respectively, while 50% of biomass accumulation was detected at 250 DAH (2700 GDDs; **Figures 2B–D**). Thus, there was an asynchrony (delay) of ∼38 days, on average, between the date when 50% of the total aboveground biomass occurred in comparison when 50% of total nutrient had accumulated.

In the partitioning process, Phase I was marked by the biomass allocation exclusively in the tops, as the occurrence of dried leaves and stalk was not visible at that growth stage (**Figure 2A**). However, an opposite trend was observed for Phases II and III, where most of the biomass was allocated in the stalk, followed by tops and dry leaves. The fraction of biomass allocated in the stalk was ∼65% of the total, which are similar toother studies, ranging from 60 to 80% (Coale et al., 1993; Inman-Bamber et al., 2002; Shukla et al., 2009; Trivelin et al., 2013). The nutrient partitioning followed the same trend as verified for biomass accumulation (**Figures 2B–D**), although a higher proportion of N, P, and K were allocated in the tops than dry leaves in the Phases II and III.

### Yield-to-Nutrient Content Relationship Sugarcane Yield

**FIGURE 3:**
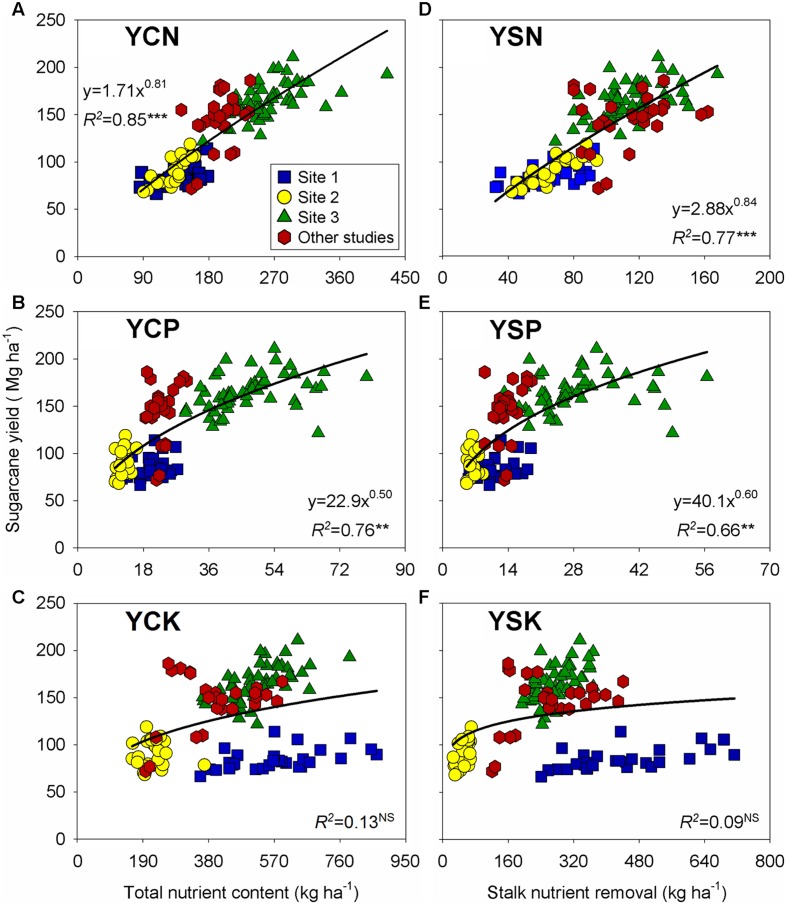
**Relationships between sugarcane yield versus aboveground nutrient content for N **(A)**, P **(B)**, and K **(C)**; and versus stalk nutrient content (nutrient removal) for N **(D)**, P **(E)**, and K **(F)**.** Each data point represents an individual field plot at sugarcane harvest of a first ratoon growing season for three site-years (*n* = 102). Red symbols were gathered from previously published documents (Shukla et al., 2009; Oliveira et al., 2010; Schultz et al., 2010; *n* = 27). ^∗∗∗^, ^∗∗^, and ^NS^: *p* ≤ 0.001, *p* ≤ 0.01, and non-significant (*p*> 0.05), respectively.

Sugarcane yield was positively related to the aboveground and stalk nutrient content, with a less than proportional change for nutrient content under high-yielding stalk observations (**Figure 3**). Both N and P showed a narrow relationship with sugarcane yield (*R*^2^ ≥ 0.66), but K presented wider variability, thus resulting in non-significant relationships (*R*^2^ ≤ 0.13; **Figures 3C,F**). For the yield-to-K relationship, Site 1 showed a range varying from 356 to 866 kg K ha^-1^ for a comparable yield level (**Figure 3C**), whereas for Site 2, K and P may have limited sugarcane yield with similar stalk K (70 kg ha^-1^) and P (9 kg ha^-1^) content, with yield ranging from 65 to 120 Mg ha^-1^ (**Figures 3E,F**).

### N:P and N:K Stoichiometry in Sugarcane Plant Components

**FIGURE 4:**
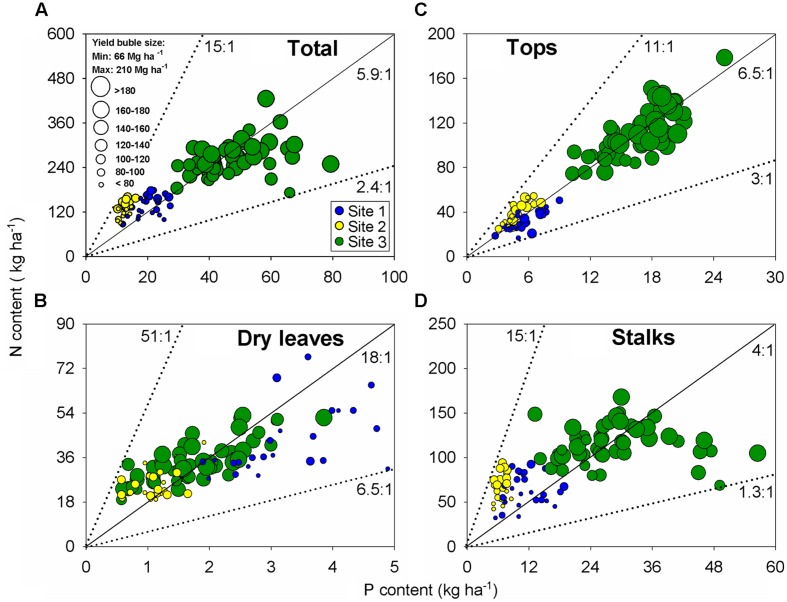
**Relationships between N versus P content for all plant components: aboveground biomass **(A)**, dry leaves **(B)**, tops **(C)**, and stalks **(D)**.** Each data point represents an individual field plot at sugarcane harvest of a first ratoon growing season for three site-years (*n* = 102). Dotted lines show boundary ratios for each component between N versus P, and black line shows the average value.

Evaluation of nutrient stoichiometry can provide an overall status of nutrient balance at a specific growth stage. Following this rationale, N:P ratio was established for all plant components (stalk, dry leaves, and tops) at harvest time (**Figure 4**). Bubble graphs portrayed N:P content association as relative to sugarcane yield (represented by bubble sizes; **Figure 4**). At the aboveground biomass level, N:P ratio presented an overall balance close to 6:1 units (**Figure 4A**), although the N:P ratio changed relative to the plant fraction: 18 units for dry leaves (**Figure 4B**), 6 unit for tops (**Figure 4C**), and 4 units for the stalk organ (**Figure 4D**). The tops followed a similar N:P ratio as documented for the aboveground plant, which exemplify the critical role of this organ for C fixation and growth/yield, with a variation from 3:1 to 11:1 (<threefold; **Figure 4C**). High-yielding sugarcane observations presented a more balanced N:P ratio (6:1 units). For example, sugarcane yield of 120 Mg ha^-1^resulted in 70 kg N ha^-1^ and 12 kg P ha^-1^ (N:P of six units) for the tops component (**Figure 4C**).

The stoichiometric evaluation was also calculated for N and K, but more variation was present than the N:P ratio. The overall N:K ratio was of 0.5:1 units, ranging from 0.1:1 to 1.2:1 (>10-fold variation; **Supplementary Figure S1**). Accordingly to the N:P ratio, the N:K stoichiometry presented a similar variation range in the tops component relative to the aboveground biomass. Detailed information about nutrient ratios (N:P and N:K) for treatments used at each site can be found in **Supplementary Table 1**.

### Efficiency Indexes on Diverse Sugarcane Yield Ranges

As expected, both biomass and nutrient content followed the yield pattern (**Table 3**). In low-yield levels, marked by yields <80 Mg ha^-1^, the aboveground biomass was of 26 Mg ha^-1^, whereas aboveground nutrient content was of 117, 16, and 233 kg ha^-1^of N, P, and K, respectively. However, under high-yield levels (>160 Mg ha^-1^), the biomass was of 89 Mg ha^-1^, while nutrient content was of 279, 50, and 560 kg ha^-1^ of N, P, and K, respectively.

**Table 3:** Sugarcane yield, aboveground biomass accumulation (ABA), aboveground nutrient content (ANC), nutrient internal efficiencies (NIE), and reciprocal internal efficiencies (RIE) of N, P, and K for diverse sugarcane yield ranges across three sites located in the Southeast region of Brazil.

Yield Mg ha^-1^	ABA Mg ha^-1^	ANC kg ha^-1^			NIE^a^ kg stalks kg^-1^ nutrient			RIE^b^ kg nutrient Mg^-1^ stalk		
				
		N	P	K	N	P	K	N	P	K
<80	26	117	16	233^c^ (381)	713	5962	478^c^ (233)	1.3	0.21	2.4^c^ (5.1)
80–100	29	141	19	244 (485)	697	5480	467 (248)	1.3	0.20	2.4 (5.4)
100–120	38	149	25	281 (333)	683	5266	439 (403)	1.4	0.23	2.5 (3.2)
120–140	60	198	33	335 (335)	664	4761	329 (329)	1.5	0.26	2.6 (2.6)
140–160	77	245	42	486 (486)	629	3750	319 (319)	1.6	0.27	3.2 (3.2)
>160	89	279	50	560 (560)	623	3649	320 (320)	1.6	0.28	3.2 (3.2)


*^a^Calculated as the sugarcane yield to the aboveground nutrient (N, P, and K) content ratio.*

*^b^Calculated as aboveground nutrient (N, P, and K) content to the sugarcane yield ratio.*

*^c^Data from Site 1 were removed from the calculation due to the high K content verified in the plant components, while values in parentheses included all sites.*

Sugarcane yield influenced all internal nutrient efficiencies for N, P, and K (**Table 3**). Maximum NIEs [713 kg stalk kg^-1^ N, 5962 kg stalk kg^-1^ P, and 478 kg stalk kg^-1^ K] occurred at lowest sugarcane yield (<80 Mg ha^-1^), while minimum NIEs [623 kg stalk kg^-1^ N, 3649 kg stalk kg^-1^ P, and 320 kg stalk kg^-1^ K] occurred at highest sugarcane yield (>160 Mg ha^-1^). Reciprocal internal efficiencies were estimated for low and high sugarcane yields (from 80 to 160 Mg ha^-1^), requiring 1.3–1.6 kg N, 0.21–0.28 kg P, and 2.4–3.2 kg K per Mg of stalk produced, respectively (**Table 3**). Oliveira (2011) reported a similar trend for RIE, with values of 1.5, 0.21, and 3.1 kg of N, P, and K, respectively.

## Discussion

The high yielding observations reported in this study were greater than previous sugarcane yield values documented by others researchers in Brazil (Gava et al., 2005; Prado and Pancelli, 2008; Oliveira, 2011; Amaral and Molin, 2013; Fortes et al., 2013; Otto et al., 2013; Rossato et al., 2013), Australia (Thorburn et al., 2011) and United States (Coale et al., 1993; McCray et al., 2014). In this study, Site 3 represents a high-yielding and productive sugarcane environment, likely explained by the combination of high soil fertility and utilization of best management practices, such as variable rate fertilizer spreader and controlled traffic through GPS guided maps. It is recognized that sugarcane yield depends on the complex interaction among genotypes, environment (soil and weather), and management practices (Thorburn et al., 2011). Several factors can contribute to the scatter and non-linear differences in yield that affect its relationship between nutrients (Setiyono et al., 2010). In this study, weather conditions, such as air temperature and precipitation, were similar for all sites (**Figure 1**), with a main difference related to the soil type and fertility status (**Table 1**). For example, in Site 1, average pH (4.3 units) and base saturation (23%) were relatively low at all evaluated soil-depth intervals, accompanied with high Al content (9.5 mmol_c_ dm^-3^) when compared to Site 3 (**Table 1**). For Site 2, the main limiting factors were K (0.3 mmol_c_ dm^-3^) and P (5.6 mg dm^-3^) levels. In summary, Sites 1 and 2 presented initial soil conditions that could constraint and severely affect sugarcane yield potential relative to Site 3. In agreement, Dias et al. (1999), documented a strong correlation between sugarcane yields and chemical properties (e.g., CEC, base saturation, exchangeable Ca, Mg, and Al, potential acidity, etc.) of subsurface horizons (25–150 cm) of six weathered soils. Treatment effect was more pronounced on Sites 1 and 2 than Site 3 (**Supplementary Table 1**), which was likely due to variations in soil chemical properties as described above.

Although the aboveground N content presented a broad variation, the overall absolute value was similar with others studies (Wood et al., 1996; Oliveira et al., 2010; Franco et al., 2011). Wood et al. (1996) documented total N content values ranging from 212 to 254 kg N ha^-1^ for several sugarcane cultivars. Franco et al. (2011) found total N content values ranging from 95 to 154 kg ha^-1^, while sugarcane yield ranged between 77 and 122 Mg ha^-1^at the same study sites (Franco et al., 2015). Oliveira et al. (2010) reported that N content ranged between 94 and 260 kg N ha^-1^, while sugarcane yield ranged from 120 to 232 Mg ha^-1^ for 11 sugarcane cultivars under irrigation in northeastern Brazil. Differences in N content could be explained by the higher yields obtained in the present study (average of 125 Mg ha^-1^). Other N sources rather than mineral fertilizer (e.g., soil N mineralization, atmospheric deposition, and biological N fixation by endophytic bacteria) can provide different amount of N for sugarcane crop (Urquiaga et al., 2012; Mariano et al., 2013; Otto et al., 2013; Vieira-Megda et al., 2015). For this study, aboveground P content presented a similar range from 21 to 39 kg P ha^-1^ as reported by several researchers (Rakkiyappan et al., 2007; Shukla et al., 2009; Oliveira et al., 2010). As related to the K content, Coale et al. (1993) and Shukla et al. (2009) reported lower values for K content removal by crop harvest of 340 and 228 kg K ha^-1^ with sugarcane yield of 96 and 80 Mg ha^-1^, respectively. For our study, high K content might have been influenced by the soil nutrient content presented at Site 1, where soil K level was ≥4.0 mmol_c_ dm^-3^ throughout the soil profile (**Table 1**). This experimental site has a long history of annual applications of vinasse and press mud. As vinasse has a high K content, amendment of this by-product might have gradually enriched soil K content (Resende et al., 2006). Thus, scientific literature is available portraying the critical role of sustaining an adequate K content for increasing sugarcane yields (Shukla et al., 2009).

Under optimal water, solar radiation, and temperature conditions, nutrient supply is the primary limiting factor affecting the process of biomass accumulation (Donaldson et al., 2008; van Heerden et al., 2010). For this study, a similar biomass accumulation pattern was previously described by others authors for plant cane and ratoon cycles for a broad range of cultivars, soil characteristics, and environmental conditions (Machado et al., 1982; Coale et al., 1993; Inman-Bamber et al., 2002; Gava et al., 2005). Phase I comprised the growth and phenology of sugarcane from emergence to 50% leaf production (half of tops already formed; **Figure 2**). The slow growth is related to the slow tillering and leaf production during plant establishment (Bell and Garside, 2005; Allison et al., 2007). Phase II is characterized by an exponential–linear biomass accumulation, consisting in leaf canopy and stalks production. After canopy establishment, the sugarcane is high efficient in converting intercepted light into biomass (Rae et al., 2005; Allison et al., 2007). The last stage (Phase III) is marked by the crop ripening and consequent sucrose accumulation in the stalk organ. Low air temperatures, reduced precipitation, and low-light photoperiod are important factors for the sugarcane ripening (Inman-Bamber et al., 2002; Cardozo and Sentelhas, 2013). At all sites, nutrients were accumulated in a linear fashion approach during Phase II as related to the potential yield production in each environment. However, in mid-point of Phase III, there was a decrease in the N and K content for all components (stalks, tops, and dry leaves; **Figure 2**). Thus, we have two hypothesis for this nutrient changes close to physiological maturity (ripening): (1) nutrient remobilization from aboveground plant components to belowground organs, such as roots and rhizomes (Andersson et al., 2005; Lim et al., 2007); and (2) specific to N allocated in the tops and dry leaves, foliar emissions of ammonia via stomata can occur during the leaf senescence, reducing the N content in plant tissues (Britto and Kronzucker, 2002; Epstein and Bloom, 2005).

Dissection of plant components for studying biomass HI is critical for understanding efficiency and potential yield for sugarcane. Inman-Bamber et al. (2002) suggested that the biomass HI (stalks to the aboveground biomass ratio) might be variable due to the amounts of straw (dry leaves and tops) recovered during the sampling procedure of sugarcane age field. In this investigation, dry leaves and tops comprised sugarcane straw, whereas tops are the plant component that accumulates the greatest amounts of nutrients (∼60% N, ∼75% P, and ∼80% K) as compared to dry leaves (**Figure 2**; **Table 2**). According to Trivelin et al. (2013), since dry leaves have low nutrient content, tops is a critical plant component that should remain as a crop residue to sustain long-term soil fertility. However, excessive stalk lodging in sugarcane fields (caused by high yields and winds) may lead to low return of the tops as straw component, since the whole plant is processed by the harvester rather than prior cutting of tops and its direct deposition on the ground. In this scenario, contribution of nutrients released from the straw for sugarcane nutrition can be less significant. From the nutrient HI results (**Table 2**), the N replacement concept (Thorburn et al., 2011), based on the fertilizer N application to replace the N removed from the system (stalk harvested), is a promising way to recommend fertilizer needs for sugarcane not only for N, but also for P and K. This approach may deliver superior environmental outcome without reducing sugarcane yield. However, as HI was not constant for any nutrient (ranging from 0.12 to 0.82, considering N, P, and K), future research on robust models for optimizing the replacement concept is needed (Setiyono et al., 2010). Low nutrient HIs for sugarcane should be considered as a better plant trait, in opposite to the biomass partitioning (HI), since more nutrients are retained in non-harvested plant components (straw) and then released to the soil through leaching (for K) and mineralization (for N and P).

Nutrient-yield relationships for N and P presented a narrowed variation for both stalks and aboveground plant relative to K variation on those plant organs (**Figure 3**). Potassium is the most important nutrient in the osmoregulation process, which is important for cell extension and stomata movement (Shukla et al., 2009; Kingston, 2014). We can speculate that the tops (functional leaves) have kept more balanced nutrient ratios for metabolic functions (Groot et al., 2003; Güsewell, 2004). Nutrient surplus (e.g., Site 1, with high K content in the soil) can be storage in other component (stalk organ), presenting a low N:K ratio (0.09:1) than tops (functional leaves; **Supplementary Figure 1**). In corn, Ciampitti and Vyn (2014) documented a more balanced N:P (5:1) and N:K (1:1) ratios under high-yielding environments. In a review analysis, Sadras (2006) showed consistent N:P ratio differences among grain crops, 4:1 for oilseed, 6:1 for cereal, and 9:1 for legume. Similar N:P ratio, 5:1 units, was synthesized by Ciampitti and Vyn (2014), in a historical and global analysis performed for corn crop. On the opposite side, dry leaves (less functional tissues) presented the largest N:P ratio, 18:1 units (ranging from 6:1 to 51:1 units; **Figure 4D**). Sugarcane yield influence was not clearly individualized for the N:P ratio of dry leaves, but lower nutrient content was observed in this plant component with a larger reduction on P (fivefold) in relation to N (twofold) when compared with the tops organ (**Figures 4B,C**). A higher N:P ratio in dry leaves is explained by the remobilization of nucleic-acid P to young leaves and the lower P demand of mature tissues (Usuda, 1995; Güsewell, 2004).

A balanced nutrient approach is critical to sustain functional tissues and to promote C fixation, growth, and increase sugar content in the sugarcane production (Allison et al., 1997, 2007; Zhao et al., 2014). From our study, nutrient content have a broad range of variability related to stalk yield levels (**Table 3**). For example, aboveground N, P, and K contents ranged from 117–280, 16–50, and 233–560 kg ha^-1^, respectively, from low (≤80 Mg ha^-1^) and high yielding sites (>160 Mg ha^-1^), respectively. Nutrient internal efficiencies can be described as the ability of a plant to transform nutrients acquired from all sources (e.g., soil, fertilizer, and atmosphere) into economic yield (Cassman et al., 2002; Dobermann, 2007; Ciampitti and Vyn, 2014), in this case represented as sugarcane yield. The higher values of NIE found in low-yielding environments represents a dilution of that nutrient in the plant, and is related to its deficiency. In contrast, low values of NIE observed in high-yielding environments represent an accumulation of that nutrient in the plant, and two hypothesis may explain this pattern, as follows: to (1) a restriction in its internal utilization due to other limiting factors; and (2) an excessive uptake (also termed as “luxury consumption”) of K, beyond the amount required for stalk production (Setiyono et al., 2010). Similar results as obtained in this investigation were reported by Setiyono et al. (2010) and Ciampitti et al. (2013) for corn, with lowest NIEs as the crop was reaching high-yield potential. The RIE values reported here are (**Table 3**) similar but higher from those documented by Silva and Casagrande (1983) and Gopalasundaram et al. (2012), which ranged from 0.56–1.20 kg N, 0.13–0.35 kg P, and 0.6–1.9 kg K per Mg of stalk produced. Lower nutrient requirements presented in previous studies are associated to low yield levels, coupled to interactions among genotype, environment, and management (Dias et al., 1999; Thorburn et al., 2011; Gopalasundaram et al., 2012). From the soil perspective, nutrient availability can limit crop acquisition, thus leading to lower biomass and stalk production (**Table 1**, **Figure 3**). In high productivity systems, fertilization of N, P, and K is essential to replenish stalk nutrient removal in order to sustainably maintain high yield levels. Therefore, soil test remains as a powerful diagnostic tool to quantify nutrient availability and subsequent fertilizer needs (McCray et al., 2014). In Brazil, however, N recommendation for sugarcane is based on the target yield concept (Cantarella et al., 1998), where soil N supply, the main source of N to the crop (Franco et al., 2011), is entirely neglected. Thus, a soil testing capable to predict *in situ* N mineralization in sugarcane fields is critically needed.

In summary, nutrient ratios can provide useful information for sugarcane growers as related to understanding crop nutrient balances and improving fertility diagnosing systems. To extend of our knowledge, this study is the first one in synthesizing data in nutrient ratios and yield potential for sugarcane crop. A main constraint on this analysis is related to when the data was collected, all at harvest time, which it did not allow the utilization of this information for in-season fertility management. A temporal analysis on nutrient ratios involving multiple in-season sampling times is needed in order to provide timely information on nutrient management recommendations for sugarcane crop. Future research should be focus on connecting soil and plant processes for identifying the main factors not only affecting nutrient ratios but also their relationship to the yield and plant biomass components.

## Conclusion

Seasonal biomass accumulation presented three growth phases (lag, exponential–linear, and stationary) during the ratoon growing season. In overall, improvement in stalk yields was tightly connected to superior aboveground biomass accumulation. Additionally, high yielding sugarcane was correlated with higher nutrient content, more balanced and narrowed nutrient ratios, and higher efficiency in producing each unit of stalk per unit of nutrient accumulated. The nutrient partitioning evaluation portrayed lower nutrient (N, P, and K) content in the dry leaves fraction but with a high N:P ratio relative to the top plant component. Greater sugarcane yield and narrowed N:P ratio (6:1) were documented for tops fraction as N and P content increased.

Stalk yield gap can be closed by better balancing nutrient ratios that can be achieved by better understanding complex plant-soil pathways related to nutrient uptake and plant utilization. This synthesis analysis not only provides a descriptive summary on the variation of nutrient ratios at harvest at varying sugarcane yield levels, but also established a foundational concept on investigating nutrient ratios by dissecting plant components. From a pragmatic standpoint, further research is needed for integrating the replacement concept in decision nutrient management support tools for assisting sugarcane producers on the farming decision-making process.

## Author Contributions

Conceived and designed the experiments: JL, IC, EM, MV-M, and PT. Performed the experiments under field conditions: JL, EM, MV-M, and PT. Performed the laboratory analyzes: JL. Contributed reagents, materials, and analysis tools: PT. Analyzed the data: JL and IC. Wrote the paper: JL, IC, EM, and PT. All the authors revised the paper.

## Conflict of Interest Statement

The authors declare that the research was conducted in the absence of any commercial or financial relationships that could be construed as a potential conflict of interest.

## Supplementary Material

**FIGURE S1 | Relationships between nitrogen (N) versus potassium (K) content for all plant components: aboveground biomass **(A)**, dry leaves **(B)**, tops **(C)**, and stalks **(D)**.** Each data point represents an individual field plot at sugarcane harvest of a first ratoon growing season for three site-years (*n* = 102). Dotted lines show boundary ratios for each component between N versus K, and black line shows the average value.

**TABLE S1 | Mean value of biomass accumulation, nutrient (N, P, and K) content, and nutrient ratios (N:P and N:K) in the stalks, dry leaves, tops, and total aboveground part of sugarcane (first ratoon cycle) across three sites located in the Southeast region of Brazil (*n* = 4 for Sites 1 and 2; *n* = 5 for Site 3).**

## Abbreviations

CEC: cation exchange capacity
DAH: days after harvest
DAP: days after planting
DRIS: diagnosis and recommendation integrated system
GDDs: growing degree days
GPS: global positioning system
HI: harvest index
HSS: humic substances solution
NIE: nutrient internal efficiency
NUE: nutrient use efficiency
RIE: reciprocal internal efficiency
