# Supplementary figures and images for: Nutrient Partitioning and Stoichiometry in Unburnt Sugarcane Ratoon at Varying Yield Levels
Source: Front Plant Sci. 2016 Apr 20;7:466. doi: 10.3389/fpls.2016.00466 (PMC4837160; doi:10.3389/fpls.2016.00466)

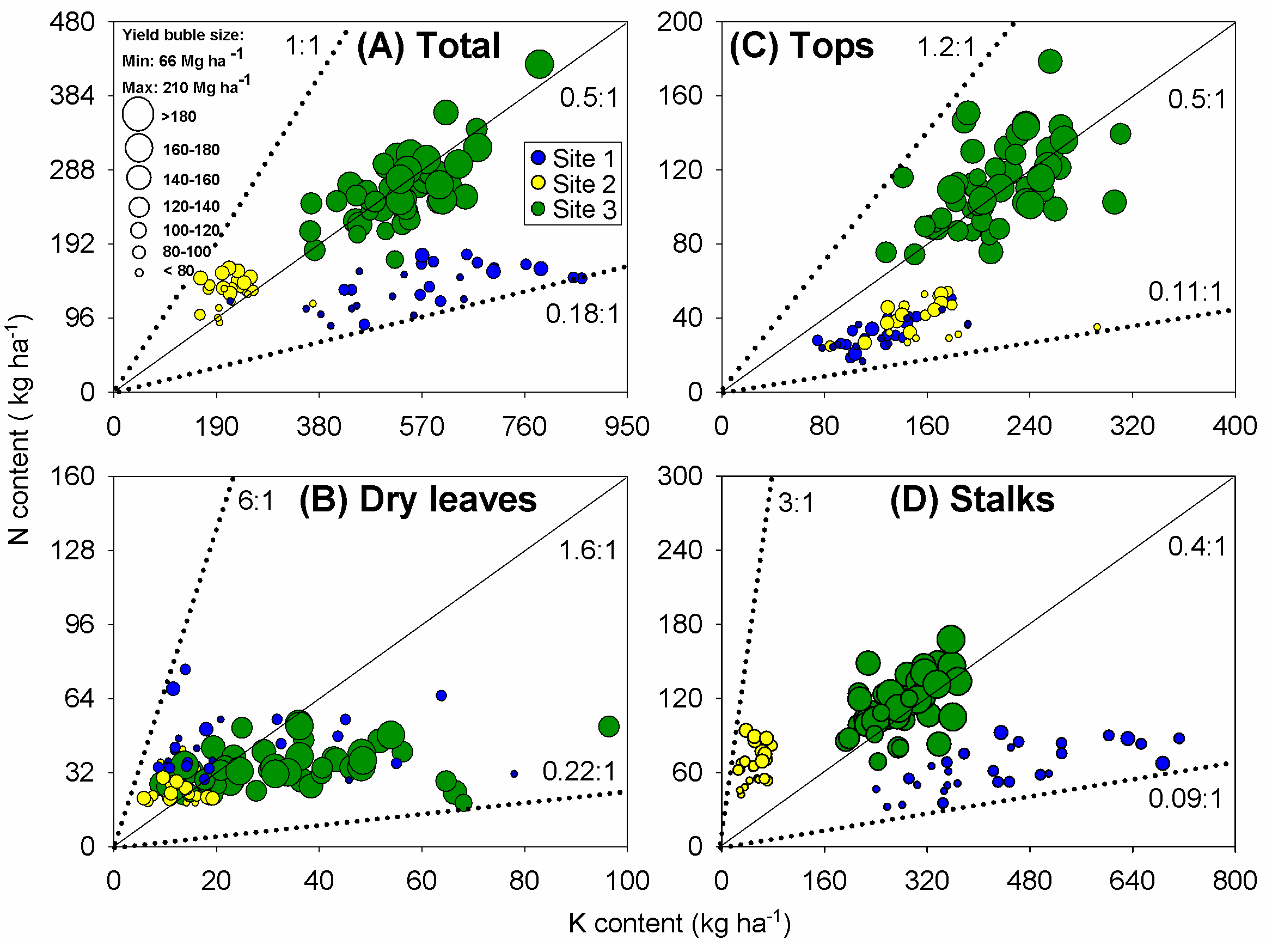

Supplement: Supplementary file 1 [file Image_1.TIF]
